# Crohn’s disease and breast cancer: a literature review of the mechanisms and treatment

**DOI:** 10.1007/s11739-023-03281-0

**Published:** 2023-05-03

**Authors:** Sisi Zhou, Jing Yu

**Affiliations:** Department of Gastroenterology, First Affiliated Hospital of Shantou University Medical College, Shantou, 515041 Guangdong Province China

**Keywords:** Crohn’s disease, Breast cancer, Signaling pathways, Cancer stem cells, TNF-α signaling, Gut flora dysbiosis, Brain–intestinal axis

## Abstract

This is a literature review describes Crohn’s disease (CD) concomitant with breast cancer and summarizes possible common pathogenic mechanisms shared by the two diseases involving the IL-17 and NF-κB signaling pathways. Inflammatory cytokines including TNF-α and Th17 cells in CD patients can induce activation of the ERK1/2, NF-κB and Bcl-2 pathways. Hub genes are involved in the generation of cancer stem cells (CSCs) and are related to inflammatory mediators, including CXCL8, IL1-β and PTGS2, which promote inflammation and breast cancer growth, metastasis, and development. CD activity is highly associated with altered intestinal microbiota processes, including secretion of complex glucose polysaccharides by Ruminococcus gnavus colonies; furthermore, γ-proteobacteria and Clostridium are associated with CD recurrence and active CD, while Ruminococcaceae, Faecococcus and Vibrio desulfuris are associated with CD remission. Intestinal microbiota disorder promotes breast cancer occurrence and development. Bacteroides fragilis can produce toxins that induce breast epithelial hyperplasia and breast cancer growth and metastasis. Gut microbiota regulation can also improve chemotherapy and immunotherapy efficacy in breast cancer treatment. Intestinal inflammation can affects the brain through the brain–gut axis, which activates the hypothalamic‒pituitary‒adrenal (HPA) axis to induce anxiety and depression in patients; these effects can inhibit the antitumor immune responses of the immune system and promote breast cancer occurrence in patients with CD. There are few studies on the treatment of patients with CD concomitant with breast cancer, but published studies show three main strategies: new biological agents combined with breast cancer treatment methods, intestinal fecal bacteria transplantation, and dietary treatment.

**Supplementary Information:**

The online version contains supplementary material available at 10.1007/s11739-023-03281-0.

## General overview of CD and breast cancer

Crohn’s disease is a chronic inflammatory disease of the gastrointestinal tract with symptoms evolving in a relapsing and remitting manner. It is also a progressive disease that leads to bowel damage and disability. All segments of the gastrointestinal tract can be affected, the most common segments include being the terminal ileum and colon. Inflammation is typically segmental, asymmetrical, and transmural. Most patients present with an inflammatory phenotype at the time of diagnosis, but over time complications (strictures, fistulas, or abscesses) will develop in half of patients, often resulting in surgery. Endoscopy remains the gold standard for diagnosis [1]. Fecal calprotectin is a helpful test that should be employed to help differentiate inflammatory bowel disease (IBD) from irritable bowel syndrome (IBS) [2]. In the postoperative setting, a fecal calprotectin concentration of more than 100 µg/g has high sensitivity for the prediction of endoscopic recurrence [1].

Cases of and studies describing CD concomitant with breast cancer are relatively rare at present. Breast cancer occurs when mammary epithelial cells proliferate out of control under the action of a variety of carcinogenic factors. During the early stage, breast cancer often manifests as a breast mass, nipple discharge, axillary lymph node enlargement or other symptoms. During the late stage, cancer cells may metastasize, directly threatening the life of patients. Breast cancer ranks first in incidence among malignant tumors occurring in females. A family history of breast cancer is an important risk factor. The risk of breast cancer is two or more times greater if the patient has a first-degree relative (mother, sister, or daughter) who has been diagnosed with breast cancer before the age of 50 years [3, 4]. Mutations in the tumor suppressor genes BRCA1 and BRCA2 are responsible for the majority of hereditary breast cancer cases. Women who inherit a BRCA1 or BRCA2 mutation have a substantial risk of developing breast cancer, which is estimated to be 72% and 69%, respectively [5]. Furthermore, patients carrying mutated BRCA1 or BRCA2 genes have a higher risk of developing other types of cancer, such as colon, prostate, pancreatic, or gastric cancer or melanoma. Therefore, identifying genes associated with breast cancer susceptibility, such as BRCA1 and BRCA2, is important for improving surveillance and developing effective preventive interventions [6]. Determination of hormone receptor status, including ER, PR, and HER2 status, is essential for newly diagnosed breast cancer patients. Such determination is crucial for choosing the most appropriate treatment option. ER status is used to identify patients with early onset breast cancer who may need tamoxifen or an aromatase inhibitor in their treatment regimen. Administration of tamoxifen as adjuvant therapy for 5 years in patients with ER + breast cancer was found to decrease recurrence by approximately 50% [7, 8].

## Molecular mechanisms shared by CD and breast cancer

CD has been identified as an independent risk factor for breast cancer development, with first-degree relatives of IBD patients being more likely to develop breast cancer [9]. Some studies have also revealed an increased risk of breast cancer in IBD patients. Breast cancer resistance protein (BCRP) is an efflux transporter that protects enterocytes from toxic compounds. Gutmann et al. found that BCRP expression was significantly reduced in patients with IBD compared with control subjects without IBD [10]. These findings suggest that inflammatory processes are responsible for the reduced levels of BCRP, which might be relevant in the pathogenesis of breast cancer [11, 12]. Japanese researchers have speculated that interleukin-1 (IL-1) polymorphisms contribute to the commonalities in genetic susceptibility to IBD and breast cancer [13]. The modification of certain genes (for example, MLH1) is associated with susceptibility to IBD and hereditary nonpolyposis colorectal cancer (HNPCC; a syndrome associated with familial inheritance of malignancy); therefore, a shift in the treatment of patients with breast malignancies towards a more targeted, immunopathological approach has been observed in recent years [14, 15].

Existing research suggests that there may be shared molecular mechanisms underlying CD and breast cancer. Publicly available microarray expression data from the Gene Expression Omnibus were analyzed, and a total of 53 overlapping differentially expressed genes (DEGs) in between the CD and breast cancer groups were identified (Table 1). These two diseases are associated with the IL-17 and NF-κB signaling pathways. Gene interaction network and module analysis demonstrated that the major hub genes were CXCL8, IL1β and PTGS2. In mononuclear and epithelial cells of the inflamed colon, inflammatory cytokines, such as TNF‑α, may activate the NF‑κB transcription factor to induce the expression of general inflammatory genes [16, 17]. When the circulating Th17 cells of patients with CD reach breast tumor tissue, Th17-cell activation may be exacerbated, and the predominance of Th1/Th17 cells leads to the upregulation of multiple cytokines, including TNF-α, IFN‑γ, IL‑1, IL‑12 and IL-17. IL1β is a factor that promotes the expansion of Th17 cells [18]. IL-17 can upregulate the expression of CXCL1 in breast cancer cells, thereby activating the AKT/NF-κB signaling pathway and promoting the growth and metastasis of breast cancer [19]. Breast tumor cells can interact with activated T cells through CD40–CD40L to increase TNF-β production and Th17 cell differentiation [20]. Th17-related inflammation may also lead to upregulation of IL17B and its receptor (IL17RB), another member of the IL-17 cytokine family, in malignant mammary epithelial cells, thereby inducing activation of the ERK1/2, NF-κB and Bcl-2 pathways and promoting inflammation and breast cancer progression [21]. Ultimately, inflammatory Th17 cells in blood in addition to NF-κB signaling may play a role in the link between inflammation, immunity, and breast tumorigenesis [22].

**Table 1:** DEGs shared by CD and breast cancer [22]

Gene symbol	Gene name	Upregulated or downregulated
AREG	Amphiregulin	Up
BAG4	BCL2associatedathanogene4	Up
BCL3	BCL3transcriptioncoactivator	Up
C5AR1	ComplementC5areceptor1	Up
CCR1	C–Cmotifchemokinereceptor1	Up
CCRL2	C–Cmotifchemokinereceptorlike2	Up
CD83	Clusterofdifferentiation83molecule	Up
CD9	Clusterofdifferentiation9molecule	Up
CXCL2	C–X–Cmotifchemokineligand2	Up
CXCL8	C–X–Cmotifchemokineligand8	Up
DNAJC3	DnaJheatshockproteinfamily(Hsp40)memberC3	Up
DSC2	Desmocollin2	Up
DUSP5	Dualspecifcityphosphatase5	Up
EGR1	Earlygrowthresponse1	Up
EGR2	Earlygrowthresponse2	Up
EGR3	Earlygrowthresponse3	Up
EPB41L3	Erythrocytemembraneproteinband4.1like3	Up
FOSB	FosBproto-oncogene,AP-1transcriptionfactorsubunit	Up
FOSL2	FOSlike2,AP-1transcriptionfactorsubunit	Up
G0S2	G0/G1switch2	Up
GAB2	GRB2associatedbindingprotein2	Up
GABARAPL1	GABAtypeAreceptorassociatedproteinlike1	Up
HBEGF	HeparinbindingEGFlikegrowthfactor	Up
IER3	Immediateearlyresponse3	Up
IL1B	Interleukin1beta	Up
MAFB	MAFbZIPtranscriptionfactorB	Up
MARCKS	MyristoylatedalaninerichproteinkinaseCsubstrate	Up
NAMPT	Nicotinamidephosphoribosyltransferase	Up
NFIL3	Nuclearfactor, interleukin3regulated	Up
NR4A2	Nuclearreceptorsubfamily4groupAmember2	Up
OSM	OncostatinM	Up
PFKFB3	6-phosphofructo-2-kinase/fructose-2,6-biphosphatase3	Up
PLAUR	Plasminogenactivator,urokinasereceptor	Up
PPP1R15A	Proteinphosphatase1regulatorysubunit15A	Up
PTGS2	Prostaglandin-endoperoxidesynthase2	Up
PTX3	Pentraxin3	Up
PVALB	Parvalbumin	Up
RAB20	RAB20,memberRASoncogenefamily	Up
RGS1	RegulatorofGproteinsignaling1	Up
SAMSN1	SAMdomain,SH3domainandnuclearlocalizationsignals1	Up
SGK1	Serum/glucocorticoidregulatedkinase1	Up
STX11	Syntaxin11	Up
TNFRSF21	TNFreceptorsuperfamilymember21	Up
TRIB1	Tribblespseudokinase1	Up
HIST2H2BE	H2Bclusteredhistone21?	Up
LOC100129518	SOD2overlappingtranscript1,SOD2	Up
ABHD17A	Abhydrolasedomaincontaining17A	Down
IFT74	Intrafagellartransport74	Down
MPHOSPH8	M-phasephosphoprotein8	Down
RBM41	RNAbindingmotifprotein41	Down
TIPRL	TORsignalingpathwayregulator	Down
NOTCH2NL	Notch2N-terminallikeA	Down
RP11-395B7.7	Pre-mRNAprocessingfactor31	Down

In addition, tumor-associated monocytes and macrophages can lead to the development of cancer stem cells (CSCs) by secreting signaling molecules (including NF-κB) [23]. NF-κB may translocate into the nucleus and subsequently activate the expression of the inflammatory genes CXCL8, IL1β and PTGS2 [24, 25]. CXCL8, IL1β and PTGS2 are inflammatory mediators that may play an important role in cancer progression by regulating CSC proliferation and self-renewal [26]. CXCL8 also has a role in neovascularization in the tumor microenvironment, which contributes to tumor growth and metastasis in the tumor microenvironment [27]. IL-β is another proinflammatory mediator that can enhance tumor-promoting inflammation in breast cancer [28]. IL-1β can activate the β-catenin signaling pathway to induce epithelial–mesenchymal transition (EMT) in breast cancer cells [29]. PTGS2-induced PGE2 production promotes the migration and EMT of human breast cancer cells [30]. Furthermore, a series of inflammatory proteins (such as CXCL8, IL1β and PTGS2) overexpressed by peripheral blood cells may increase systemic inflammation in female patients with CD [31].

## The intestinal inflammatory response and TNF-α signaling in CD promote the development of breast cancer

**Fig. 1:**
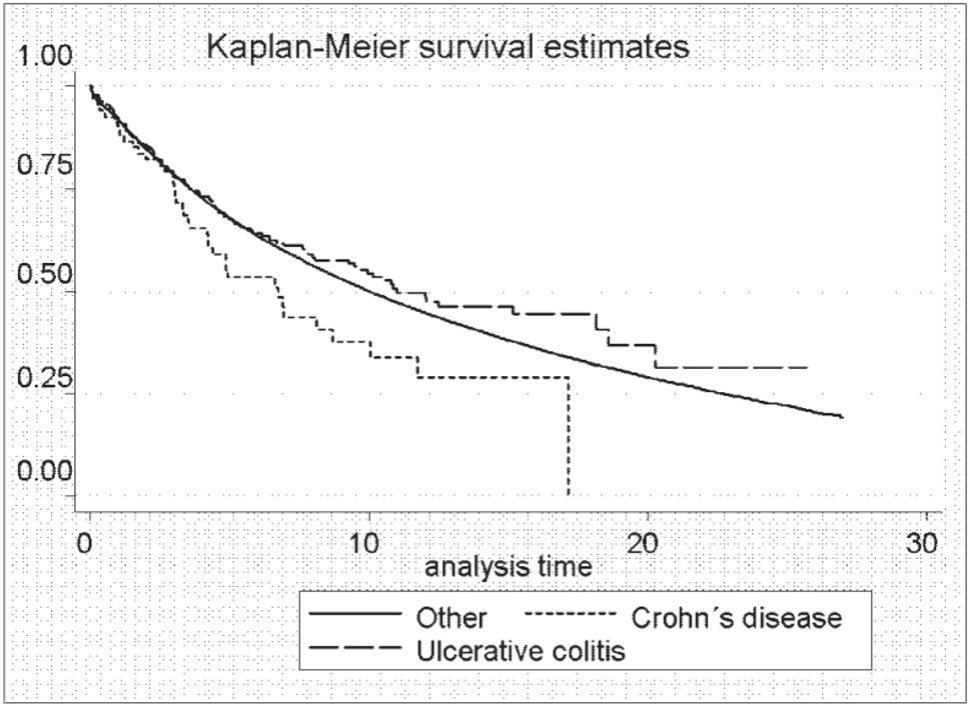
Shows that survival at 5 years and 10 years was lower in cancer patients with CD than in cancer patients without IBD: 52% vs. 67% and 38% vs. 50%, respectively. [33]

The development of intestinal inflammation in CD involves intestinal epithelial cells, neutrophils, macrophages, dendritic cells, natural killer cells, lymphocytes and other cellular components that make up the intestinal immune system. CD4 + T cells are the main lymphocytes in enteritis tissues, and the inflammatory process is induced by Th1 or Th2 cells [32]. In a retrospective study conducted in Denmark, patients had breast cancer that was staged as local, regional, distant and unknown. However, CD is not clearly staged. CD patients with local, regional, distant, or unknown stage breast cancer had higher mortality rates than cancer patients without IBD (Fig. 1). Patients with CD had a more advanced stage of breast cancer than cancer patients without IBD. In the adjusted analysis stratified by receipt of radiotherapy and chemotherapy, receipt of chemotherapy was associated with a poorer prognosis in cancer patients with CD than in cancer patients without IBD (MRR 1.93; 95% CI 1.00–3.72) (data not presented). The authors speculated that treatment with chemotherapy may be accompanied by serious side effects, such as extensive ulcerations of the intestines, and the disruption of mucosal integrity in addition to neutropenia can result in sepsis. Therefore, this process may represent the active phase of CD. There has been no clear study of the survival rate of patients at each stage of chemotherapy for breast cancer relative to the active stage or remission stage of CD. The survival rates of patients treated with chemotherapy with four different stages of breast cancer who also had active CD or were in CD remission were not clearly shown in the study [33]. This result suggests that the systemic inflammation associated with IBD may be involved in the malignant progression of breast cancer. Recently, several studies have shown that TNF-α promotes metastasis and is involved in EMT, which is necessary for tumor cell migration to establish metastasis [34]. Active TNF-α consists of a homologous trimer that continuously transitions into TNF-α monomers. Its dissociation rate and binding affinity are independent of the TNF-α concentration, but high concentrations of TNF-α play an important role in maintaining biological activity. Conversion between TNF-α monomers and trimers is blocked by treatment with TNF-α inhibitors, such as adalimumab, infliximab, etanercept, golimumab and cetuximab [35]. TNF-α treatment has been reported to inhibit proliferation and induce apoptosis in some breast cancer cell lines [36]. In particular, prolonged exposure to TNF-α in breast cancer cell lines induces upregulation of the transcriptional repressor Twist1 through activation of IKKβ and NF-κB, induces EMT, and enhances CSC properties [37]. In a study of a cohort of patients with inflammatory breast cancer, a direct correlation between TNF-α production by peripheral blood T lymphocytes and the detection of circulating tumor cells expressing EMT markers was found [38].

## Gut flora dysbiosis promotes the development of CD and breast cancer

Changes in the human intestinal microbiome are associated with host disease. One research group cultured R. gnavus colonies in the laboratory and found that R. gnavus synthesizes and secretes complex glucose polysaccharides with rhamnose skeletons and glucose side chains, which can effectively induce dendritic cells to secrete inflammatory cytokines (such as TNF-α). TNF-α secretion depends on Toll-like receptor 4 (TLR4). Identifying increased polysaccharide gene expression before CD worsens will provide a valuable reference for future research [39]. The first randomized controlled trial (RCT) of CD suggested that CD recurrence was associated with failure of fecal microbiota transplantation (FMT). CD recurrence was associated with γ-proteobacteria and Clostridia, which include Ruminococcus species, such as Ruminococcus faecalis, while Ruminococcus desulfuris was associated with remission [40]. Metagenomic data from 857 patients from various regions of the world (China, the United States, The Netherlands, Spain) revealed that DL-endopeptidase gene expression was significantly reduced in CD patients and negatively correlated with disease activity. DL-Endopeptidase is a key enzyme involved in NOD2 ligand production, and intestinal flora disorder is also an important factor affecting NOD2 signal transduction. These findings suggest that NOD2 activation may be an important mechanism affecting the efficacy of traditional probiotics, such as Lactobacillus salivus [41].

One study found a strong relationship between the health of the intestinal flora and the development of breast cancer in mice. Environmental changes in the intestinal flora triggered an inflammatory response in the mice that altered the growth trend of breast cancer cells. Disrupting intestinal flora homeostasis in ER-positive cancer-bearing mice with antibiotics induced a systemic inflammatory response, and tumor spread was observed. Furthermore, homeostasis of the intestinal flora is related to inflammation and can even promote the metastasis of tumor cells [42]. In addition, the intestinal flora affects resistance to breast cancer chemotherapy. Through studies of HER2-positive breast cancer mouse models and patients, it was found that the intestinal microbiota directly affects the efficacy of trastuzumab, and fecal bacteria transplanted from FMT-R mice into HER2 + breast cancer mice reproduced the patient response to trastuzumab. The β-diversity of fecal flora, which is known as intersample diversity, was found to be positively correlated with immune signals associated with IFN, IL12-NO, activated CD4 + T cells, and dendritic cells. These results suggest that regulation of the intestinal flora can optimize trastuzumab treatment, so the intestinal flora has the potential to be a marker of therapeutic response [43]. Cancer Discovery recently published a paper comparing the microbiotas of the normal breast and breast cancer and revealed the presence of Bacteroidetes fragilis in breast cancer. Enterotoxigenic Bacteroides fragilis (ETBF) in the breast and intestines can rapidly secrete Bacteroides fragilis toxin (BFT) and induce breast epithelial hyperplasia, which increases the growth and metastasis of breast cancer. BFT induces morphological and functional changes in normal mammary epithelial cells and breast cancer cells, enhancing stem cell characteristics and promoting the development of multifocal mammary tumors. In addition, short-term exposure to culprit Bacteroides species induces long-term “BFT memory” which is mediated by β-catenin and Notch1 signaling [44].

## CD promotes the development of breast cancer through the brain–intestinal and is axis related to anxiety and depression

Chronic psychological stress has been found to be associated with gastrointestinal dysfunction. Chronic psychosocial stress has also been identified as a risk factor for the development and subsequent recurrence of IBD [45]. There are different degrees of stress in patients with persistent CD or breast cancer alone, while CD patients with breast cancer may experience considerably more stress. However, recent research has revealed that stress, as an external stimulus, can inhibit the antitumor effect of the body's immune system [46].

As there is no cure for CD, its symptoms can persist for life. Patients with IBD have been reported to be at high risk for social isolation due to the stress associated with the disease, negative patient perception of social support systems and social stigma resulting from lack of public awareness. In one study with a mean follow-up period of 11.84 years, Cox proportional risk assessment showed that IBD patients who were socially isolated had a 69% increased risk of premature death compared with IBD/CD/ulcerative colitis (UC) patients who were not socially isolated, and this risk was more strongly associated with the CD subtype [47]. In the meta-IVW analysis, gene-predicted IBD was associated with depression [48]. The gastrointestinal tract is innervated by the central nervous system(CNS), the autonomic nervous system (ANS), and the enteric nervous system (ENS). Neuroregulation of the gastrointestinal tract can be divided into four levels. The first level is the regulation mediated by the ENS itself. The ENS includes the intermuscular nerve plexus and submucosal nerve plexus, which have a certain degree of autonomous regulation and are relatively independent of the CNS. The second level is the prevertebral ganglia, which receive information from the CNS and ENS. The third is the sympathetic and parasympathetic nerves afferent to the gastrointestinal tract; Level 4 involves the more advanced cortical center [49]. Intestinal inflammation can affect the brain through the brain–gut axis, which connects the gastrointestinal tract with the CNS. The CNS and gut microbes interact with each other through the endocrine and immune systems. Signals from the gut affect brain function and thus affect emotional, behavioral and cognitive functions [50–52]. Research has shown that psychological factors interact with the ANS and ENS through the hypothalamic‒pituitary‒adrenal (HPA) axis, thus affecting the intestinal inflammatory response and immune process [53].

Stress can induce the hypothalamus to produce corticotropin releasing hormone (CRH) which acts on the pituitary gland to produce adrenocorticotropic hormone (ACTH) and then induce the adrenal gland to produce cortisol, which enters the blood circulation and directly affecting the gastrointestinal tract [54]. Previous studies revealed that the cingulate gyrus, insula, amygdala and thalamus were significantly activated in patients with CD. Subsequent activation of the hippocampus, prefrontal cortex, and secondary somatosensory cortex confirmed that patients with CD had affected sensory, cognitive, and emotional production [55]. There may be a common molecular mechanism shared by CD and breast cancer that may be related to the IL-17 and NF-κB signaling pathways. A synergistic effect of IL-17 and IL-22 has been demonstrated to enhance the expression of certain inflammatory cytokines in mucosal immune responses. Epinephrine amplifies the effect of IL-17A on chemokine expression and neutrophil migration, suggesting a synergistic role of stress-related hormones and IL-17 in promoting the recruitment and transport of neutrophils in IBD [56]. A recent study showed that prolactin induced by psychological stress increases IL-17 production in regulatory T cells [57].

The results demonstrated a clear positive association between the presence of multimorbidity and the risk of depression in survivors of breast cancer. The elevated risk of depression could be related to higher treatment burden, increasing disability and lower quality of life associated with having multiple health conditions. Multimorbidity may also cause additional health care costs and financial burdens, which may adversely affect mental health among cancer survivors [58]. In the absence of a laboratory or imaging test, eliciting patient symptoms by clinical interview or a self-report scale is the main way to detect depression and monitor its response to treatment. The PHQ-9 has generally been shown to be similar or superior in performance to competing depression scales, including in special populations such as older adults, adolescents, pregnant or postpartum women, diverse racial/ethnic groups, and patients with various medical and psychiatric diseases and across clinical settings. Its nine items comprise the DSM criteria for depressive disorders, making it both a severity and potentially diagnostic measure. Therefore, it is recommended to give a preliminary diagnosis using the PHQ-9 scoring scale when diagnosing the disease or observing patients with depression, slow thinking and reduced volitional activity [59] (Table 2).

**Table 2:** Correlation between Crohn's disease and breast cancer and depression

Depression	Study		Results	References
Crohn’s disease	Article	Unadjusted model	HR (95% CI) 1.67 (1.44–1.93)	[60]
		Multivariable adjusted model	HR (95% CI) 2.11 (1.65–2.70)	
Crohn’s disease	Review		95% CI 20.7–30.0 *P* < 0.0001	[61]
Crohn’s disease	Meta-analysis	Any	Pooled prevalence (%, 95% CI) 24.8% (20.7–29.3) *P* < 0.0001	[62]
		HADS	Pooled prevalence (%, 95% CI) 23.0% (18.8–27.6) *P* < 0.0001	
		PHQ-9	Pooled prevalence (%, 95% CI) 29.0% (18.8–40.3) *P* < 0.0001	
Crohn’s disease	Article		OR (95% CI) 0.37 (0.044–3.18)	[63]
Crohn’s disease	Review	HBI	RR(95% CI) 2.3 (1.9–2.3)	[64]
Crohn’s disease	Meta-analysis	DeLange	OR(95% CI)1.27 [1.02; 1.58]	[48]
		FinnGen	OR(95% CI)1.36 [0.94; 1.98]	
		Combined effect	OR(95% CI)1.29 [1.07; 1.56)	
Crohn’s disease	Article	Age-adjusted	HR (95% CI)2.55 (1.51–4.29) *P* value 0.0003	[65]
		Multivariate	HR (95% CI) 2.36 (1.40–3.99) *P* value 0.0010	
Breast cancer	Meta-analysis	NO.study5	Before diagnosis HR (95% CI) 1.33 (1.22–1.45)	[66]
			After diagnosis HR (95% CI) 1.22 (1.05–1.41)	
Breast cancer	Meta-analysis	Major depression	Unadjusted HR (95% CI) 1.00 (0.88–1.15)	[67]
		Minor depression	Unadjusted HR (95% CI) 0.92 (0.82–1.02)	
Breast cancer	Article	Married	OR (95%CI) 0.27 (0.11–0.63) *P* value 0.002	[68]
		Inactive	OR (95%CI) 0.46 (0.23–0.92) *P* value 0.029	

## Current treatment measures

### Combination of novel biologic agents with existing breast cancer and other cancer treatment methods

The use of TNF-α blockers in the treatment of breast cancer is relatively unexplored. TNF-α blockers are currently recommended for patients with IBD who have been diagnosed with cancer within the past 5 years [69]. On one hand, only one clinical trial has shown etanercept to be safe and well-tolerated in breast cancer patients, but etanercept has not been found to produce an objective disease response, which may be due to advanced disease stage [70]. On the other hand, the continued use of methotrexate, thiopurine, and TNF-α blockers in patients with cancer and IBD does not increase the risk of new cancer development or exacerbate existing cancer [71]. Another study of patients with rheumatoid arthritis and breast cancer showed that TNF-α blockade did not increase breast cancer recurrence compared to other treatments [72]. Considering all of these results, TNF-α blockers are a promising tool for treating breast cancer patients in combination with existing cancer treatments [73].

WA meta-analysis of multiple studies showed that both ustekinumab (UST) and vedolizumab (VDZ) were associated with a low tumor risk [74]. Studies suggest that VDZ should be used in patients with lymphoma and cancer of other origins, such as skin malignancies or solid tumors, but not in patients with lymphoma of gastrointestinal origin, in whom VDZ should be used with caution [75]. The GEMINI LTS study, VDZ postmarket safety data and multiple recent studies suggest that VDZ does not increase the risk of new or recurrent malignancies in patients with prior tumors with or without IBD [76–78]. In a multivariate Cox analysis, after adjusting for age, IBD subtype, smoking status, cancer recurrence risk, and cancer stage, there was no increase in subsequent cancer risk found to be associated with VDZ treatment [79].

A review of previous clinical trials concluded that patients with a history of leukemia, lymphoma, nonmelanoma lymphoma, melanoma, solid tumors, urinary tumors, or cervical cancer could be treated with UST [80]. P40 monomers, members of the IL-12 family, have tumor-promoting effects, and IL-23 is directly and indirectly involved in the genesis, growth and metastasis of tumor cells. Ustekinumab reduces the risk of tumors by antagonizing IL-12/IL-23 and does not increase the risk of tumors in elderly patients [81–83]. UST was also found not to increase tumor risk in patients in a phase III IM-UNITI clinical trial [84]. In addition to TNF-α blockers and VDZ, UST therapy should be considered after a thorough evaluation of the tumor nature and risk of recurrence in patients with common primary tumor types or a history of common tumors, even in patients with severe disease [85, 86]. However, the safety risks and efficacy need to be weighed when using UST. If a patient has a solid tumor, lymphoma or melanoma and is receiving chemotherapy or radiotherapy, the use of UST should be suspended, but if the patient is not receiving chemotherapy or radiotherapy, continued UST use may be appropriate. Patients with nonmelanoma skin cancer can receive UST regardless of radiotherapy or chemotherapy status. The benefits and risks should be analyzed according to the clinical situation [74, 87] (Table 3).

**Table 3:** Correlation between cancer and biological agents, particularly breast cancer

Biological agent	Cancer	Results		References
Vedolizumab	Solid Tumor	Continue		[75]
	Lymphoma			
	Breast cancer	Ratio of expected to observed (95% CI)	0.397 (0.048–1.435)	[76]
	Gynaecologic cancer		0.821 (0.021–4.575)	
	Lymphoma		0.309 (0.008–1.722)	
	Incident cancer	Multivariate analysis HR [95% CI]	0.2 [0.1–0.6] *P* = 0.01	[77]
	New or recurrent cancer	Adjusted HR (95% CI) for incident cancer	0.72 (0.38–1.39)	[78]
	New or recurrent cancer	Adjusted HR (95% CI) for incident cancer	1.36 (0.27–7.01)	[79]
	Solid cancers	Consider using		[80]
	Cervical cancer			
	Solid tumor	Continue		[87]
	Continue	Continue		[85]
	Primary cancer diagnosis	HR (95% CI) for incident cancer	0.18 (0.03–1.35) *P* = 0.096	[101]
Ustekinumab	New or recurrent cancer	Adjusted HR (95% CI) for incident cancer	0.96 (0.17–5.41)	[79]
	Solid cancers	Consider using		[80]
	Cervical cancer			
	Breast cancer	Continue		[85]
	Primary cancer diagnosis	Primary cancer diagnosis	0.88 (0.25–3.03) *P* = 0.833	[101]
Anti-TNF	Incident cancer	Multivariate analysis HR [95% CI]	0.4 [0.2–0.8] *P* = 0.01	[77]
	New or recurrent cancer	Adjusted HR (95% CI) for incident cancer	1.03 (0.65–1.64)	[78]
	New or recurrent cancer	Adjusted HR (95% CI) for incident cancer	0.70 (0.10–4.74)	[79]
	Solid cancers	Consider using		[80]
	Cervical cancer			
	Breast cancer	Continue		[85]
	Risk of colorectal cancer	OR (95% CI)	0.69 (0.66–0.73) *P* < 0.0001	[100]
	Primary cancer diagnosis	HR (95% CI) for incident cancer	0.47 (0.20–1.12) *P* = 0.087	[101]

Cytotoxic chemotherapy for breast cancer may induce and maintain CD remission by causing cell death or preventing the division of rapidly dividing cells, such as T lymphocytes or malignant cells, resulting in anticancer and immunosuppressive effects [88].

### FMT for patients with CD with breast cancer

Intestinal bacteria can migrate to nonmucosal sites and colonize parenteral tumors in steady state conditions to form a tumor-specific flora that has diagnostic and prognostic value and affects the occurrence and development of tumors, regulating antitumor immunity and the effect of cancer therapy [89]. When a patient has severe microbiota imbalance and intestinal inflammation, they also have a high fecal calprotectin level, suggesting that we can predict the response to treatment based on a patient's intestinal microbiota composition [90]. Although the pathogenesis of CD is different, FMT may be an effective treatment in which healthy flora are transferred to CD patients to restore the balance between the host and the microbiota. In a pilot study, transplantation of a healthy donor microbiota was associated with maintenance of CD remission [91]. The research teams at the Universities of Wurzburg and Marburg have successfully demonstrated for the first time that microbial metabolites can increase the cytotoxic activity of some immune cells and positively influence the efficacy of tumor therapy. It has long been suggested that the intestinal flora can directly modulate the effects of specific tumor immunotherapies, among which immune the effects of checkpoint inhibitor (ICI) therapy and cytotoxic T lymphocyte (CTL) “CD8 + T”-mediated adoptive T-cell therapy (TIL-ACT) are particularly closely related to the intestinal flora [92]. FMT improved the immunotherapy response in treatment-resistant patients in early clinical trials published in Science [93]. Subsequently, the gene expression patterns of regulatory T (Treg) cells in the colon lamina propria of Bifidobacterium- and PBS-treated mice were analyzed, and the results showed that Bifidobacterium treatment increased the expression of inflammatory genes, such as II10ro, Cxcr5 and II17ra. Flow cytometry experiments confirmed the increased expression of IL-10R and IL-10 in Treg cells in the colon lamina propria after Bifidobacterium treatment. The remission of enteritis induced by Bifidobacterium treatment was completely eliminated in mice with II-10 gene knockout and IL-22 blockade, suggesting that IL-22 and IL-10 are necessary for Bifidobacterium treatment to improve intestinal immunopathology.

Bifidobacterium treatment significantly reduces the side effects of tumor immunotherapy by significantly reducing the expression of specific immune checkpoints and enhancing that of others. These results will aid the optimization of relevant clinical immunotherapy strategies and lay a solid theoretical foundation for improving the structure of the intestinal flora using probiotics and enhancing the comprehensive effect of tumor immunotherapy [94].

### Dietary treatment

The Crohn’s disease exclusion diet (CDED) is recommended for patients with CD and can maintain intestinal flora homeostasis and ensure the health of the intestinal environment. Good dietary habits combined with exercise, adequate sleep and consumption of high-fiber foods can help prevent breast cancer. The CDED is a combination of enteral nutrition and whole-grain-restrictive diets. The diet is low in fat and animal protein and rich in compound carbohydrates and dietary fiber. It does not include gluten, dairy, and certain food additives, such as emulsifiers, maltodextrins, carrageenan and sulfites. The CDED induces beneficial changes in the fecal microbiome and the course of the disease, reducing the abundance of bacteria with proinflammatory activity and increasing the abundance of anti-inflammatory bacteria [95]. Moreover, fiber, ω-3 polyunsaturated fatty acids (PUFAs), vitamins C and E, fruits and vegetables may have a protective role by reducing oxidative stress and lowering chronic inflammation [96]. A previous study found that the CDED combined with partial enteral nutrition (CDED + PEN) can alleviate childhood CD illness and reduce intestinal inflammation. This combination may reduce the relative abundance of Proteobacteria, especially Escherichia coli, and increase the production of the anti-inflammatory metabolite SCFAs in the intestinal mucosa. The results of a prospective, RCT showed that CDED + PEN induced clinical remission of CD in children by correcting intestinal microbial imbalance and improving intestinal metabolite composition [97]. The heavy consumption of vegetables and fruits mediated by the Mediterranean diet provides considerable amounts of both polyphenols and fiber, both of which have been suggested to prevent carcinogenesis [98].

## Conclusion

CD is a chronic recurrent disease. It occurs more frequently in young people, and the onset is insidious. During the early stage, it manifests as only intermittent abdominal discomfort or is asymptomatic. CD can occur in any part of the digestive tract but mainly affects the end of the ileum and colon; later, it can later seriously interfere with intestinal functions and cause irreversible damage. Breast cancer is one of the most common malignant tumors in females, and the main causes of death in patients are postoperative recurrence and metastasis despite continuous advances in various therapeutic methods, including surgery, radiotherapy, chemotherapy and immunotherapy, and significant improvements in patient survival and the overall survival rate [99]. Cases of CD concomitant with breast cancer and related studies are relatively rare at present. Furthermore, research regarding common pathogenic mechanisms shared by the two diseases has not provided clear conclusions.

Genetic modification undoubtedly plays a crucial role in both diseases. The IL-17 and NF-κB signaling pathways have been studied as common mechanisms shared by the two diseases. In addition to the inflammatory characteristics of the intestinal tract in IBD, the activation of inflammatory cytokines such as TNF-α and Th17 cells in breast tissue leads to the upregulation of various cytokines, including TNF-α, IFN-γ, IL-1, IL-12 and IL-17; these changes promote the upregulation of IL17B and its receptor (IL17RB) in malignant mammary epithelial cells and the expression of CXCL1 in breast cancer cells, thus activating the AKT/NF-κB, ERK1/2, NF-κB and Bcl-2 signaling pathways and promoting inflammation and the growth, metastasis, and development of breast cancer. Research on key pathways and genes may help us better understand why and how CD contributes to the development of breast cancer.

Hub genes are important in peripheral blood cells and are involved in the generation of CSCs. CXCL8, IL1β and PTGS2 are inflammatory mediators that regulate CSC proliferation and self-renewal. CXCL8 plays a role in angiogenesis, and IL-1β enhances tumor inflammation, activates the β-catenin signaling pathway and induces EMT in breast cancer cells in addition to the expression of PTGS2. PTGS2 expression is induced by PGE2 and the inflammatory cytokine TNF-α, promoting the development of breast cancer in patients with CD. Therefore, TNF-α blockers combined with cancer therapies may be beneficial in the treatment of patients with CD and breast cancer. Drugs that promote or may promote tumor development should be avoided in IBD patients with a history of tumors. The introduction of novel biologics, such as VDZ and UST, provides additional options. Many studies suggest that UST and VDZ can be used for the treatment of patients with common primary tumor types or a previous history of common tumors or even patients with malignant tumors and severe disease. VDZ should be used with caution in the treatment of primary gastrointestinal lymphoma. However, cytotoxic chemotherapy for breast cancer does not aggravate CD; in contrast, it can maintain CD remission by inducing cell death.

The gut contains the greatest variety and number of microbes in the human body. The normal intestinal microbial community is generally dynamically balanced, which maintains the normal immune and defense functions of the body, protects the intestinal epithelium, and reduces inflammatory reactions and the occurrence of malignant tumors. Patients with CD and patients with breast cancer have varying degrees of intestinal flora disorder. Although research on the differences in microflora between CD and breast cancer is relatively lacking, FMT has not only shown obvious efficacy in the treatment of IBD but can also improve tumor immunotherapy efficacy and prognosis. The CDED, which is rich in complex carbohydrates and dietary fiber, combined with enteral nutrition and a whole-grain-restrictive diet has been shown to have beneficial effects on the fecal microbiome and reduce the number of proinflammatory active bacteria to alleviate CD and reduce intestinal inflammation; it can also prevent breast cancer. Fecal bacteria imbalance should be monitored in patients with breast cancer and CD. Oral capsule FMT therapy combined with existing cancer treatment methods can be used instead of invasive operations, but patient psychology and relevant therapies also need to be considered for patients with breast cancer and CD.

**Fig. 2:**
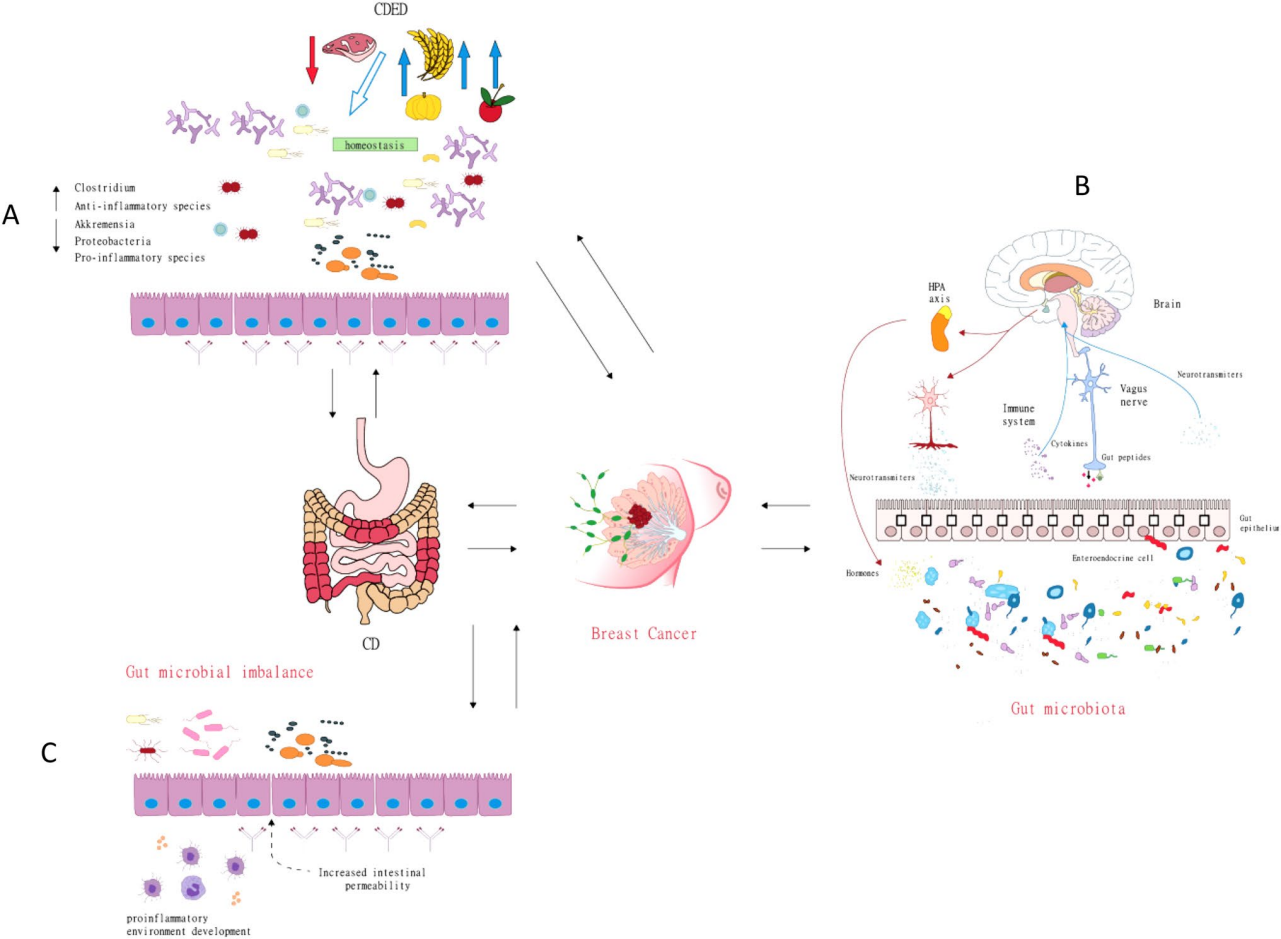
**A** Shows that the CDED diet is low in fat and animal protein and rich in compound carbohydrates and dietary fiber. It does not include gluten, dairy, and certain food additives, such as emulsifiers, maltodextrins, carrageenan, and sulfites. In the second period, a fixed portion of whole-grain bread is allowed, as are small amounts of nuts, fruits, legumes, and vegetables. Patients with dietary strictures continue the quantitative restriction of fruits and vegetables on an individual basis. **B** Shows that the intestinal inflammatory response can induce anxiety and depression in patients through the reverse effect of the brain–gut axis, such as changing the blood‒brain barrier permeability to induce a central inflammatory response, activating local brain regions, and activating the hypothalamus–pituitary–adrenal (HPA) axis. **C** Shows the gut microbial imbalance and development of CD. Intestinal microbial imbalance can destroy the tight connection between intestinal wall mucosal cells, increase intestinal permeability, and promote the development of inflammation [95].

Long-term psychological stress can enhance the secretion of IL-17 to promote the recruitment and transport of neutrophils and inhibit the antitumor effect of the immune system in IBD. As CD is an IBD, the intestinal inflammatory response in CD can activate the HPA axis through opposite effects of the brain–intestine axis, thus inducing anxiety and depression in patients. Multiple studies have reported that breast cancer patients also have different severities of anxiety and depression. Therefore, the disease mechanisms and mental and emotional influences need to be further studied in patients with CD concomitant with breast cancer, which may provide insights regarding the link between the development of CD and breast cancer. If necessary, a multidisciplinary team should be formed to choose ideal treatment and prevention measures. Moreover, patients should be encouraged to seek support to maintain a good mental state and keep their bodies and minds healthy (Fig. 2, Table 3).

## Supplementary Information

Below is the link to the electronic supplementary material.

Supplementary file1 (DOCX 840 KB)

## Data Availability

Data sharing not applicable—no new data generated; data sharing is not applicable to this article as no new data were created or analyzed in this study.
