# Supplementary material for: Crohn’s disease and breast cancer: a literature review of the mechanisms and treatment
Source: Intern Emerg Med. 2023 May 3;18(5):1303–16. doi: 10.1007/s11739-023-03281-0 (PMC10412481; doi:10.1007/s11739-023-03281-0)
Supplement: Supplementary file 1 — Supplementary file1 (DOCX 840 KB) [file 11739_2023_3281_MOESM1_ESM.docx]

Response:Thanks for your comment.

Reviewer #2:

1) The pages are not numbered (at least in my version), which makes the work of the reviewer not easy.

Response 1 : Article has been revised.

2) The definition of Crohn's disease (CD) given at the beginning of the article is misleading. I would recommend Lancet 2017; 389: 1741-55.

Response in the page 2: Crohn’s disease is a chronic inflammatory disease of the gastrointestinal tract with symptoms evolving in a relapsing and remitting manner. It is also a progressive disease that leads to bowel damage and disability. All segments of the gastrointestinal tract can be affected, the most common segments include being the terminal ileum and colon. Inflammation is typically segmental, asymmetrical, and transmural. Most patients present with an inflammatory phenotype at diagnosis, but over time complications (strictures, fistulas, or abscesses) will develop in half of patients, often resulting in surgery.Endoscopy remains the gold standard for diagnosis. [1]Fecal calprotectin is a helpful test that should be employed to help differentiate inflammatory bowel disease (IBD) from irritable bowel syndrome (IBS).[2]In the postoperative setting, a faecal calprotectin concentration of more than 100 µg/g has high sensitivity for prediction of endoscopic recurrence.[1]

References:

[1] Torres J, Mehandru S, Colombel JF, Peyrin-Biroulet L. Crohn's disease. Lancet. 2017;389(10080):1741-1755. doi:10.1016/S0140-6736(16)31711-1
[2] Lichtenstein GR, Loftus EV, Isaacs KL, Regueiro MD, Gerson LB, Sands BE. ACG Clinical Guideline: Management of Crohn's Disease in Adults [published correction appears in Am J Gastroenterol. 2018 Jul;113(7):1101]. Am J Gastroenterol. 2018;113(4):481-517. doi:10.1038/ajg.2018.27
3) Several sentences (e.g., "The specific aetiology of CD is not clear, but it is believed to be caused by a combination of various factors, including geographic factors, changes in the intestinal flora, disordered and poor immune function and damage to the epithelial barrier"; or "Cases of and studies describing CD concomitant with breast cancer are relatively rare at present") in several sections of the manuscript lack of references.

Response in the page 3: The sentence "The specific aetiology of CD is not clear, but it is believed to be caused by a combination of various factors, including geographic factors, changes in the intestinal flora, disordered and poor immune function and damage to the epithelial barrier" has since been deleted.

There is no reference for this sentence "Cases of and studies describing CD concomitant with breast cancer are relatively rare at present" .

4) The section of the manuscript concerning the relationship between biological therapy and the risk of malignancy in general, and breast cancer in particular, deserves to be expanded with an approach that takes into account the all-available evidence especially for each molecule currently on the market.

Response in the page 26 : **Table 3：Correlation between cancer and biological agents, particularly breast cancer.**

There are still few correlational studies between biologics and cancer. There are many types of cancer, such as prostate cancer, lung cancer, and colorectal cancer. The list has been shortened to reduce the length of the article, and most cancer classification and biologics relationships are consistent. Therefore, this paper mainly lists the correlation between biological agents and gynecological diseases and cancers. (Table 3)

**Table 3：Correlation between cancer and biological agents, particularly breast cancer.**

| **Biological agent** | **cancer** | **Results** | | | | **References** |
| --- | --- | --- | --- | --- | --- | --- |
| Vedolizumab | Solid Tumor | Continue | | | | [75] |
|  | Lymphoma |  |  |  |  |  |
|  | Breast cancer | Ratio of expected to observed（95%CI） | 0.397（0.048-1.435） | | | [76] |
|  | Gynaecologic cancer |  | 0.821 (0.021–4.575) | | |  |
|  | Lymphoma |  | 0.309 (0.008–1.722) | | |  |
|  | incident cancer | Multivariate analysis HR [95% CI] | 0.2 [0.1-0.6]P=0.01 | | | [77] |
|  | new or recurrent cancer | adjusted HR (95% CI) for incident cancer | 0.72 (0.38–1.39) | | | [78] |
|  | New or Recurrent Cance | adjusted HR (95% CI) for incident cancer | 1.36 (0.27-7.01) | | | [79] |
|  | Solid cancers | Consider using | | | | [80] |
|  | cervical cancer |  |  |  |  |  |
|  | Solid tumor | Continue | | | | [87] |
|  | primary cancer diagnosis | HR (95% CI) for incident cancer | | | 0.18 （0.03–1.35） P=0.096 | [101] |
|  | Breast cancer | Continue | | | | [102] |
| Ustekinumab | new or recurrent cancer | adjusted HR (95% CI) for incident cancer | | 0.96 (0.17-5.41) | | [79] |
|  | Solid cancers | Consider using | | | | [80] |
|  | cervical cancer |  |  |  |  |  |
|  | primary cancer diagnosis | primary cancer diagnosis | | 0.88 （0.25–3.03） P= 0.833 | | [101] |
|  | Breast cancer | Continue | | | | [102] |
| Anti-TNF | incident cancer | Multivariate analysis HR [95% CI] | | 0.4 [0.2-0.8]P=0.01 | | [77] |
|  | new or recurrent cancer | adjusted HR (95% CI) for incident cancer | | 1.03 (0.65–1.64) | | [78] |
|  | new or recurrent cancer | adjusted HR (95% CI) for incident cancer | | 0.70 (0.10-4.74) | | [79] |
|  | Solid cancers | Consider using | | | | [80] |
|  | cervical cancer |  |  |  |  |  |
|  | Risk of colorectal cancer | OR (95% CI) | | 0.69（0.66-0.73）P<0.0001 | | [100] |
|  | primary cancer diagnosis | HR (95% CI) for incident cancer | | 0.47（0.20–1.12）  P= 0.087 | | [101] |
|  | Breast cancer | Continue | | | | [102] |

References:

75.Click B, Regueiro M. A Practical Guide to the Safety and Monitoring of New IBD Therapies. *Inflamm Bowel Dis*. 2019;25(5):831-842. doi:10.1093/ibd/izy313

76.Card T, Ungaro R, Bhayat F, Blake A, Hantsbarger G, Travis S. Vedolizumab use is not associated with increased malignancy incidence: GEMINI LTS study results and post-marketing data. *Aliment Pharmacol Ther*. 2020;51(1):149-157. doi:10.1111/apt.15538

77.Poullenot F, Amiot A, Nachury M, et al. Comparative Risk of Incident Cancer in Patients with Inflammatory Bowel Disease with Prior Non-digestive Malignancy According to Immunomodulator: a Multicentre Cohort Study. *J Crohns Colitis*. 2022;16(10):1523-1530. doi:10.1093/ecco-jcc/jjac061

78.Vedamurthy A, Gangasani N, Ananthakrishnan AN. Vedolizumab or Tumor Necrosis Factor Antagonist Use and Risk of New or Recurrent Cancer in Patients With Inflammatory Bowel Disease With Prior Malignancy: A Retrospective Cohort Study. *Clin Gastroenterol Hepatol*. 2022;20(1):88-95. doi:10.1016/j.cgh.2020.10.007

79.Hong SJ, Zenger C, Pecoriello J, et al. Ustekinumab and Vedolizumab Are Not Associated With Subsequent Cancer in IBD Patients with Prior Malignancy [published online ahead of print, 2022 Mar 9]. *Inflamm Bowel Dis*. 2022;izac035. doi:10.1093/ibd/izac035

80.Lin SC, Goldowsky A, Papamichael K, Cheifetz AS. The Treatment of Inflammatory Bowel Disease in Patients With a History of Malignancy. *Inflamm Bowel Dis*. 2019;25(6):998-1005. doi:10.1093/ibd/izy376

87.Click B, Regueiro M. Managing Risks with Biologics. *Curr Gastroenterol Rep*. 2019;21(2):1. Published 2019 Jan 11. doi:10.1007/s11894-019-0669-6

100.Alkhayyat M, Abureesh M, Gill A, et al. Lower Rates of Colorectal Cancer in Patients With Inflammatory Bowel Disease Using Anti-TNF Therapy. Inflamm Bowel Dis. 2021;27(7):1052-1060. doi:10.1093/ibd/izaa252

101.Hasan B, Tandon KS, Miret R, et al. Ustekinumab does not increase risk of new or recurrent cancer in inflammatory bowel disease patients with prior malignancy. J Gastroenterol Hepatol. 2022;37(6):1016-1021. doi:10.1111/jgh.15806

102.Beaugerie L, Rahier JF, Kirchgesner J. Predicting, Preventing, and Managing Treatment-Related Complications in Patients With Inflammatory Bowel Diseases. Clin Gastroenterol Hepatol. 2020;18(6):1324-1335.e2. doi:10.1016/j.cgh.2020.02.009

5) On what is probably page 8 the Authors write 'A cohort study in Denmark found that patients with breast cancer and CD who received chemotherapy had a shorter survival than those with breast cancer alone who received chemotherapy'. This statement needs further clarification: at what stage were breast cancer patients? What was the severity of the CD?

Response in the page 8:This is a retrospective study.Information on breast cancer patients (stage and treatment) diagnosed between 1980 and 2004 was sourced from the Danish Cancer Registry.The stage of breast cancer is local, regional,distant and unknown stage.However,crohn's disease is not clearly staged.

Crude mortality rates in the whole follow-up period were statistically higher in patients with CD (105 per 1000 person years [PY]; 95% CI 77–144). CD Patients with local, regional, distant, or unknown stage breast cancer had higher mortality rates than their without Inflammatory Bowel Disease.Patients with CD had more advanced stage of breast cancer than patients without IBD.In the adjusted analysis stratified by receipt of radiotherapy and chemotherapy, receipt of chemotherapy was associated with a poorer prognosis in patients with CD compared to patients without IBD (MRR 1.93; 95% CI 1.00-3.72) (data not presented).Authors think treatment with chemotherapy may be explained by serious side effects like extensive ulcerations of the intestines; the disruption of mucosal integrity together with neutropenia can result in sepsis.So this is supposed to be the active phase of Crohn's disease.There is no clear study of the survival rate of each stage of chemotherapy for breast cancer relative to the active stage or remission stage of Crohn's disease.The survival rates of chemotherapy at four different stages of breast cancer, respectively in active Crohn's disease or remission, were not clearly shown in the paper.

The figure 1 shows that survival at 5 years and 10 years was lower in cancer patients with CD than in cancer patients without IBD: 52% versus 67% and 38% versus 50%, respectively. [33]

Reference:

[33]Søgaard KK, Cronin-Fenton DP, Pedersen L, Sørensen HT, Lash TL. Survival in Danish patients with breast cancer and inflammatory bowel disease: a nationwide cohort study. Inflamm Bowel Dis. 2008;14(4):519-525. doi:10.1002/ibd.20341

6)The correlation between CD, breast cancer and depression need to be discussed more clearly: for example, was depression assessed before or after diagnosis of either disease?

Response in the page 12:

**CD promotes the development of breast cancer through the brain-intestinal and is axis related to anxiety and depression**

Chronic psychological stress has been found to be associated with gastrointestinal dysfunction. Chronic psychosocial stress has also been identified as a risk factor for the development and subsequent recurrence of IBD [45]. There are different degrees of stress in patients with persistent CD or breast cancer alone, while CD patients with breast cancer may experience considerably more stress. However, recent research has revealed that stress, as an external stimulus, can inhibit the antitumor effect of the body's immune system[46].

As there is no cure for CD, its symptoms can persist for life. Patients with IBD have been reported to be at high risk for social isolation due to the stress associated with the disease, negative patient perception of social support systems and social stigma resulting from lack of public awareness. In one study with a mean follow-up period of 11.84 years, Cox proportional risk assessment showed that IBD patients who were socially isolated had a 69% increased risk of premature death compared with IBD/CD/ulcerative colitis (UC) patients who were not socially isolated, and this risk was more strongly associated with the CD subtype[47]. In the meta-IVW analysis, gene-predicted IBD was associated with depression[48]. The gastrointestinal tract is innervated by the central nervous system(CNS), the autonomic nervous system(ANS), and the enteric nervous system(ENS).Neuroregulation of the gastrointestinal tract can be divided into four levels.The first level is the regulation mediated by the ENS itself. The ENS includes the intermuscular nerve plexus and submucosal nerve plexus, which have a certain degree of autonomous regulation and are relatively independent of the CNS. The second level is the prevertebral ganglia, which receive information from the CNS and ENS. The third is the sympathetic and parasympathetic nerves afferent to the gastrointestinal tract; Level 4 involves the more advanced cortical center [49]. Intestinal inflammation can affect the brain through the brain-gut axis, which connects the gastrointestinal tract with the CNS. The CNS and gut microbes interact with each other through the endocrine and immune systems. Signals from the gut affect brain function and thus affect emotional, behavioral and cognitive functions [50-52]. Research has shown that psychological factors interact with the ANS and ENS through the hypothalamic‒pituitary‒adrenal (HPA) axis, thus affecting the intestinal inflammatory response and immune process [53].

Stress can induce the hypothalamus to produce corticotropin releasing hormone(CRH)which acts on the pituitary gland to produce adrenocorticotropic hormone (ACTH) and then induce the adrenal gland to produce cortisol, which enters the blood circulation and directly affecting the gastrointestinal tract[54].Previous studies revealed that the cingulate gyrus, insula, amygdala and thalamus were significantly activated in patients with CD. Subsequent activation of the hippocampus, prefrontal cortex, and secondary somatosensory cortex confirmed that patients with CD had affected sensory, cognitive, and emotional production [55]. There may be a common molecular mechanism shared by CD and breast cancer that may be related to the IL-17 and NF-κB signaling pathways. A synergistic effect of IL-17 and IL-22 has been demonstrated to enhance the expression of certain inflammatory cytokines in mucosal immune responses. Epinephrine amplifies the effect of IL-17A on chemokine expression and neutrophil migration, suggesting a synergistic role of stress-related hormones and IL-17 in promoting the recruitment and transport of neutrophils in IBD[56].A recent study showed that prolactin induced by psychological stress increases IL-17 production in regulatory T cells[57].

The results demonstrated a clear positive association between the presence of multimorbidity and the risk of depression in survivors of breast cancer. The elevated risk of depression could be related to higher treatment burden, increasing disability and lower quality of life associated with having multiple health conditions. Multimorbidity may also cause additional health care costs and financial burdens, which may adversely affect mental health among cancer survivors[58].In the absence of a laboratory or imaging test, eliciting patient symptoms by clinical interview or a self-report scale is the main way to detect depression and monitor its response to treatment. The PHQ-9 has generally been shown to be similar or superior in performance to competing depression scales, including in special populations such as older adults, adolescents, pregnant or postpartum women, diverse racial/ethnic groups, and patients with various medical and psychiatric diseases and across clinical settings. Its nine items comprise the DSM criteria for depressive disorders, making it both a severity and potentially diagnostic measure. Therefore, it is recommended to give a preliminary diagnosis using the PHQ-9 scoring scale when diagnosing the disease or observing patients with depression, slow thinking and reduced volitional activity [59].

References:

[45]Mawdsley JE, Rampton DS. Psychological stress in IBD: new insights into pathogenic and therapeutic implications. Gut. 2005;54(10):1481-1491. doi:10.1136/gut.2005.064261

[46]Häuser W, Janke KH, Klump B, Hinz A. Anxiety and depression in patients with inflammatory bowel disease: comparisons with chronic liver disease patients and the general population. Inflamm Bowel Dis. 2011;17(2):621-632. doi:10.1002/ibd.21346

[47]Chen J, Geng J, Wang J, et al. Associations between inflammatory bowel disease, social isolation, and mortality: evidence from a longitudinal cohort study. Therap Adv Gastroenterol. 2022;15:17562848221127474. Published 2022 Sep 30. doi:10.1177/17562848221127474

[48]Luo J, Xu Z, Noordam R, van Heemst D, Li-Gao R. Depression and Inflammatory Bowel Disease: A Bidirectional Two-sample Mendelian Randomization Study. J Crohns Colitis. 2022;16(4):633-642. doi:10.1093/ecco-jcc/jjab191

[49]Mulak A, Bonaz B. Irritable bowel syndrome: a model of the brain-gut interactions. Med Sci Monit. 2004;10(4):RA55-RA62.

[50]Sidebottom AM, Rodriguez TG, Karpin JE, Rubin DT. Clinical and Translational Considerations for Understanding Depression and Anxiety in Patients with Inflammatory Bowel Disease. Gastroenterol Res Pract. 2021;2021:6689443. Published 2021 Mar 5. doi:10.1155/2021/6689443

[51]Sinagra E, Utzeri E, Morreale GC, Fabbri C, Pace F, Anderloni A. Microbiota-gut-brain axis and its affect inflammatory bowel disease: Pathophysiological concepts and insights for clinicians. World J Clin Cases. 2020;8(6):1013-1025. doi:10.12998/wjcc.v8.i6.1013

[52]Moulton CD, Pavlidis P, Norton C, et al. Depressive symptoms in inflammatory bowel disease: an extraintestinal manifestation of inflammation?. Clin Exp Immunol. 2019;197(3):308-318. doi:10.1111/cei.13276

[53]Li XJ, You XY, Wang CY, et al. Bidirectional Brain-gut-microbiota Axis in increased intestinal permeability induced by central nervous system injury. CNS Neurosci Ther. 2020;26(8):783-790. doi:10.1111/cns.13401

[54]Abautret-Daly Á, Dempsey E, Parra-Blanco A, Medina C, Harkin A. Gut-brain actions underlying comorbid anxiety and depression associated with inflammatory bowel disease. Acta Neuropsychiatr. 2018;30(5):275-296. doi:10.1017/neu.2017.3

[55]Rubio A, Pellissier S, Van Oudenhove L, et al. Brain responses to uncertainty about upcoming rectal discomfort in quiescent Crohn's disease - a fMRI study. Neurogastroenterol Motil. 2016;28(9):1419-1432. doi:10.1111/nmo.12844

[56]Deng Q, Chen H, Liu Y, et al. Psychological stress promotes neutrophil infiltration in colon tissue through adrenergic signaling in DSS-induced colitis model. Brain Behav Immun. 2016;57:243-254. doi:10.1016/j.bbi.2016.04.017

[57]Wu W, Sun M, Zhang HP, et al. Prolactin mediates psychological stress-induced dysfunction of regulatory T cells to facilitate intestinal inflammation [published correction appears in Gut. 2020 Oct;69(10):e7]. Gut. 2014;63(12):1883-1892. doi:10.1136/gutjnl-2013-306083

[58] Foster M, Niedzwiedz CL. Associations between multimorbidity and depression among breast cancer survivors within the UK Biobank cohort: a cross-sectional study. BMC Cancer. 2021;21(1):650. Published 2021 May 31.

doi:10.1186/s12885-021-08409-z

[59]Kroenke K. PHQ-9: global uptake of a depression scale. World Psychiatry. 2021;20(1):135-136. doi:10.1002/wps.20821

7) Table 1: Please insert the legend for 'DEGs'.

Response in the page 6:

| **Gene symbol** | **Gene name** | **Upregulated or downregulated** |
| --- | --- | --- |
| AREG | Amphiregulin | Up |
| BAG4 | BCL2associatedathanogene4 | Up |
| BCL3 | BCL3transcriptioncoactivator | Up |
| C5AR1 | ComplementC5areceptor1 | Up |
| CCR1 | C‑Cmotifchemokinereceptor1 | Up |
| CCRL2 | C‑Cmotifchemokinereceptorlike2 | Up |
| CD83 | Clusterofdifferentiation83molecule | Up |
| CD9 | Clusterofdifferentiation9molecule | Up |
| CXCL2 | C‑X‑Cmotifchemokineligand2 | Up |
| CXCL8 | C‑X‑Cmotifchemokineligand8 | Up |
| DNAJC3 | DnaJheatshockproteinfamily(Hsp40)memberC3 | Up |
| DSC2 | Desmocollin2 | Up |
| DUSP5 | Dualspecifcityphosphatase5 | Up |
| EGR1 | Earlygrowthresponse1 | Up |
| EGR2 | Earlygrowthresponse2 | Up |
| EGR3 | Earlygrowthresponse3 | Up |
| EPB41L3 | Erythrocytemembraneproteinband4.1like3 | Up |
| FOSB | FosBproto‑oncogene,AP‑1transcriptionfactorsubunit | Up |
| FOSL2 | FOSlike2,AP‑1transcriptionfactorsubunit | Up |
| G0S2 | G0/G1switch2 | Up |
| GAB2 | GRB2associatedbindingprotein2 | Up |
| GABARAPL1 | GABAtypeAreceptorassociatedproteinlike1 | Up |
| HBEGF | HeparinbindingEGFlikegrowthfactor | Up |
| IER3 | Immediateearlyresponse3 | Up |
| IL1B | Interleukin1beta | Up |
| MAFB | MAFbZIPtranscriptionfactorB | Up |
| MARCKS | MyristoylatedalaninerichproteinkinaseCsubstrate | Up |
| NAMPT | Nicotinamidephosphoribosyltransferase | Up |
| NFIL3 | Nuclearfactor,interleukin3regulated | Up |
| NR4A2 | Nuclearreceptorsubfamily4groupAmember2 | Up |
| OSM | OncostatinM | Up |
| PFKFB3 | 6‑phosphofructo‑2‑kinase/fructose‑2,6‑biphosphatase3 | Up |
| PLAUR | Plasminogenactivator,urokinasereceptor | Up |
| PPP1R15A | Proteinphosphatase1regulatorysubunit15A | Up |
| PTGS2 | Prostaglandin‑endoperoxidesynthase2 | Up |
| PTX3 | Pentraxin3 | Up |
| PVALB | Parvalbumin | Up |
| RAB20 | RAB20,memberRASoncogenefamily | Up |
| RGS1 | RegulatorofGproteinsignaling1 | Up |
| SAMSN1 | SAMdomain,SH3domainandnuclearlocalizationsignals1 | Up |
| SGK1 | Serum/glucocorticoidregulatedkinase1 | Up |
| STX11 | Syntaxin11 | Up |
| TNFRSF21 | TNFreceptorsuperfamilymember21 | Up |
| TRIB1 | Tribblespseudokinase1 | Up |
| HIST2H2BE | H2Bclusteredhistone21? | Up |
| LOC100129518 | SOD2overlappingtranscript1,SOD2 | Up |
| ABHD17A | Abhydrolasedomaincontaining17A | Down |
| IFT74 | Intrafagellartransport74 | Down |
| MPHOSPH8 | M‑phasephosphoprotein8 | Down |
| RBM41 | RNAbindingmotifprotein41 | Down |
| TIPRL | TORsignalingpathwayregulator | Down |
| NOTCH2NL | Notch2N‑terminallikeA | Down |
| RP11‑395B7.7 | Pre‑mRNAprocessingfactor31 | Down |

References:[22]Zhou J, Yang R. Identification of key pathways and genes shared between Crohn's disease and breast cancer using bioinformatics analysis. Oncol Lett. 2020;20(4):119. doi:10.3892/ol.2020.11981

8) The images in Figure 2 are low resolution: it would be appropriate for the images to be high resolution. Furthermore, the figures need a clear explanation of their meaning.

Figure 2B

Figure 2C

Figure 2A

Response in the page 21:Figure 2A shows that the CDED diet is low in fat and animal protein and rich in compound carbohydrates and dietary fiber. It does not include gluten, dairy, and certain food additives, such as emulsifiers, maltodextrins, carrageenan, and sulfites. In the second period, a fixed portion of whole-grain bread is allowed, as are small amounts of nuts, fruits, legumes, and vegetables. Patients with dietary strictures continue the quantitative restriction of fruits and vegetables on an individual basis. Figure 2B shows that the intestinal inflammatory response can induce anxiety and depression in patients through the reverse effect of the brain-gut axis, such as changing the blood‒brain barrier permeability to induce a central inflammatory response, activating local brain regions, and activating the hypothalamus-pituitary-adrenal (HPA) axis. Figure 2C shows the gut microbial imbalance and development of CD. Intestinal microbial imbalance can destroy the tight connection between intestinal wall mucosal cells, increase intestinal permeability, and promote the development of inflammation.

References:95.Starz E, Wzorek K, Folwarski M, et al. The Modification of the Gut Microbiota via Selected Specific Diets in Patients with Crohn's Disease. Nutrients. 2021;13(7):2125. Published 2021 Jun 22. doi:10.3390/nu13072125
